# The Dynamics Between Responses to Aging Restrictions and Day-to-Day Functioning as a Key to Successful Aging

**DOI:** 10.3390/bs15091153

**Published:** 2025-08-25

**Authors:** Michal Tsadok-Cohen, Sara Rosenblum, Ortal Cohen Elimelech, Simona Ferrante, Sonya Meyer

**Affiliations:** 1 Department of Occupational Therapy, Faculty of Social Welfare and Health Sciences, University of Haifa, Haifa 3498838, Israel; rosens@univ.haifa.ac.il (S.R.); ocoheneli@staff.haifa.ac.il (O.C.E.); 2 Department of Electronics, Information and Bioengineering, Politecnico di Milano, 20133 Milano, Italy; simona.ferrante@polimi.it; 3 Department of Occupational Therapy, Faculty of Health Sciences, Ariel University, Ariel 4070000, Israel; sonyam@ariel.ac.il

**Keywords:** acceptance, positive mindset, self-efficacy, coping strategy, occupational adaptation, successful aging

## Abstract

Age-related physiological and cognitive changes significantly affect older adults’ participation in day-to-day functioning. This interview study aimed to uncover and illuminate the intricate dynamics between individuals’ responses to aging restrictions and day-to-day functioning, and how they relate to successful aging. We used a qualitative research design to explore the various responses to aging decline and their implications for daily functioning among older adults. Eighteen in-depth interviews were conducted with older adults, focusing on their occupational characteristics, needs, and responses to aging constraints. The transcripts were analyzed using principles of constructivist grounded theory. Three main categories were identified regarding older adults’ responses to the decline in abilities that come with age: (a) *acceptance*, reflecting the individual’s ability to adapt to the age-related changes and constraints; (b) *personal resources*, including a *positive mindset* and *self-efficacy*; and (c) *coping strategies*, including *meaningful roles* and *occupational adaptation*. This study’s findings indicate three types of responses to aging restrictions that may contribute to greater engagement in daily life and, consequently, be a key to successful aging. Developing individually tailored interventions that focus on occupational adaptations according to individual needs and preferences is vital in helping older adults maintain their daily functioning and quality of life.

## 1. Introduction

The global population is aging much more rapidly than it has in the past. The proportion of the world’s population over 60 years is expected to nearly double between 2015 and 2050 (World Health Organization, 2018). This phenomenon raises concerns about older adults’ health and quality of life. Aging has long been associated with chronic health conditions, and older adults often suffer from multimorbidity. Age-related physiological changes can lead to difficulty performing activities, cognitive impairment, and other functional decreases (Bay et al., 2023). Age-related changes in resources may include sensory decline (e.g., hearing/vision loss), cognitive decline (e.g., memory difficulties), and motor decline (e.g., more frequent falls; Bay et al., 2023; Harada et al., 2013).

The changing resources and constraints have far-reaching consequences for older adults’ functioning while participating in daily activities. For example, neurodegenerative changes decrease *executive functions*, higher-level cognitive abilities that enable goal-directed behavior (Ferguson et al., 2021). This decline can affect the ability to live independently and perform complex functions such as shopping, driving, and managing money (Adrian et al., 2011; Harada et al., 2013; Rosenblum et al., 2013; Suchy et al., 2023). Other health declines, including vision problems, medical illnesses, and neurologic or musculoskeletal disorders, may influence driving (Falkenstein et al., 2020; Harada et al., 2013). Aging processes also affect the ability to process sensory information adequately (T. S. Kim & Chung, 2013). Poorer sensory-processing abilities are correlated with reduced occupational participation in daily life, such as engaging in social and leisure activities (Engel-Yeger & Rosenblum, 2017). Given the fundamental implications of these changing resources and the constraints on day-to-day functioning, it is crucial to understand the various responses and coping strategies older adults use in these incidences towards successful aging.

Although the term *successful aging* is widely used, it lacks a universal definition or standardized measure (Menassa et al., 2023). A common definition describes successful aging as a multidimensional concept with three components: absence of illness or disability associated with illness, preserved physical and cognitive abilities, and participation in social and productive activities. Although the first two components are crucial for successful aging, only their combination with life engagement leads to a complete successful aging experience (Rowe & Kahn, 1997). Thus, the components of day-to-day functional activities and participation in varied life domains among older adults must be considered. An additional approach to successful aging defines it as a process of adapting to the new biological and environmental demands associated with growing older (Baltes & Baltes, 1990). This approach emphasizes the personal cognitive strategies that older people use to age successfully. Moreover, a positive psychological adaptation in later life was identified as an important component of successful aging (S. H. Kim & Park, 2017). Other definitions of successful aging include identifying cognitive plasticity as a factor related to successful aging (Navarro & Calero, 2018). Moreover, dependency risk, as reflected in deficits in activities of daily living, is identified as one of the characteristics of successful aging (Mount et al., 2019). Beyond these definitions, research shows that responding flexibly to age-related transitions is vital to successful aging (Feliciano et al., 2022).

One example of such adaptive response is *acceptance.* A fundamental prerequisite for successful aging is accepting one’s limitations or losses (Jenull et al., 2023). Research indicates that older adults strive to accept the aging process and value enjoying life despite the challenges of aging (Reich et al., 2020)., Specifically, Kishita et al. (2016) demonstrated the effectiveness of acceptance interventions in physical and mental illness during aging. The positive effects were also described in improved flexibility and coping with stressors (Roberts & Sedley, 2016). Although several studies have referred to this acceptance, none addressed its implications for daily functioning. Further research is still needed to understand how acceptance promotes participation in daily activities, such as volunteering or engaging in social activities.

Additional responses to age-related changes are optimism and a sense of self-efficacy. Optimism may promote life engagement and assist in coping with stressors, such as the declining resources that accompany aging. A recent cross-cultural systematic review attempted to trace older adults’ perspectives on successful aging. It found that maintaining a positive attitude was related to how older adults managed physical limitations. Even when older adults had objectively poor health, they overcame that limitation with a positive attitude (Reich et al., 2020). Similarly, O’Connor and Graham (2019) reported that optimism predicts a longer lifespan among older adults, regardless of their health status. Optimism has been linked to life satisfaction (Dumitrache et al., 2019) and positive mental and physical health outcomes (Achat et al., 2000). It is a protective factor against the harmful effects of activity limitations on life satisfaction over time (Cheng et al., 2022). The literature on optimism during aging typically concentrates on its effects on life expectancy and health outcomes rather than the relationship between optimism and the individual’s participation in day-to-day activities. Besides optimism, previous studies indicated that personal resources such as self-esteem and self-efficacy buffer the negative impact of activity limitations on well-being (Jonker et al., 2009). *Self-efficacy* refers to a person’s perceptions of their capacity to perform a specific function successfully (Bandura, 1977). Whereas a low sense of self-efficacy has been related to depression, anxiety, and helplessness, high self-efficacy is associated with successful aging and helping to initiate and maintain healthy behaviors (Araújo et al., 2016; Remm et al., 2021; Schwarzer & Fuchs, 1996). Self-efficacy is essential in all stages of life, but especially during old age, because it influences this age group’s beliefs, emotions, and behaviors (Lozoya et al., 2022). As people age, they encounter a more comprehensive range of personal and social circumstances that challenge their sense of control and independence. Studies consistently show that older adults with high self-efficacy manage chronic diseases more effectively and adhere better to medication regimens (e.g., Náfrádi et al., 2017). Together, these resources, acceptance, optimism, and self-efficacy, may shape how older adults navigate age-related constraints and maintain participation. However, more research is needed to uncover the relationships between these responses, older adults’ day-to-day functioning, and successful aging.

*Successful aging* definitions usually tend to reflect each investigator’s viewpoint. Yet, a profound understanding of older adults’ subjective perception remains scarce (Teater & Chonody, 2020). Subjectivity and personal psychological goals are essential to directing a person’s behavior (Levitt-Frank & Shoshana, 2023). It is valuable for contextualizing and enriching the research process and its products, can add depth to research findings, and help situate the research within relevant social contexts (Gough & Madill, 2012). Specifically, Nakagawa et al. (2024) found that self-perceptions of aging change over time. Moreover, a focus group study explored older adults’ perceptions of aging well. It stressed the importance of listening to older adults because the state of *aging well* refers to their subjective feelings and depends on personal views (Halaweh et al., 2018). The significance of qualitative research is increasingly acknowledged for its contribution to comprehending and representing diverse, often-unrecognized perspectives (Godfrey, 2015). Given that the concept of aging well relies on subjective and personal perspectives (Halaweh et al., 2018), we found a qualitative study design to be appropriate for exploring it.

A few qualitative studies noted interesting findings about older adults’ viewpoints on successful aging (e.g., Betlej, 2023; Burton et al., 2024; Kusumaningrum et al., 2025). One interview study emphasized the significance of life satisfaction, supportive environments, and social integration in successful aging (Betlej, 2023). A second focus group study indicated that psychosocial factors, such as positive attitude and adaptability, are more critical than disease, disability, or function (Reichstadt et al., 2007). Lastly, interviews with older adults found that they perceive successful aging “as a balance between self-acceptance and self-contentedness on one hand and engagement with life and self-growth in later life on the other” (Reichstadt et al., 2010, p. 567). These studies applied various qualitative approaches (including narrative, phenomenological, and content analysis methods). The present study also used a qualitative approach consisting of interviews with older adults. However, it applied a constructivist grounded theory approach, resulting in the development of a conceptual model.

Our recent qualitative study highlighted older adults’ diverse expectations and attitudes. It emphasized the significance of engaging with life and self-management abilities in the context of successful aging (Tsadok-Cohen et al., 2023). The study’s results also touched on the dynamics between the individual’s responses to aging restrictions and day-to-day functioning. Building on those findings, the current interview study aimed (a) to identify how older adults respond to age-related restrictions and (b) to examine how these responses influence their day-to-day participation and perceptions of successful aging.

## 2. Materials and Methods

### 2.1. Design

We used a qualitative study design. Semistructured interviews were undertaken to identify various responses to aging decline and their implications for day-to-day functioning among older adults. The interviews took place from February 2021 to July 2022, partially characterized bylockdowns due to COVID-19.

### 2.2. Setting and Participants

This study was part of a large project (ESSENCE) funded by the European Union’s Horizon 2020 research and innovation program (grant agreement no. 101016112). The University of Haifa Ethics Committee provided ethical approval (086/21). This interview study builds on the authors’ previous findings (Tsadok-Cohen et al., 2023). All interviews were conducted in Hebrew online (via Zoom videoconferencing software version 5.5.0, Zoom Video Communications, Inc., San Jose, CA, USA) except for one conducted face-to-face. The interviewees provided online informed consent before participating. Interviews were video- and audio-recorded, transcribed, and chosen quotations were translated into English by the interviewer. The inclusion of video facilitated transcription by considering non-verbal cues such as facial expressions, gestures, and body language to refine the contextual meaning. The data were securely stored electronically on a password-protected computer after the lead author de-identified the transcripts and assigned pseudonyms. Moreover, to uphold confidentiality and anonymity, any potentially identifiable information was eliminated from the selected quotes, and the original records were deleted after transcription.

The sample included 18 independent older adults aged 65 years or older (*M*_age_ = 72.7 years, *SD* = 5.31) living at home or in assisted living facilities and able to use a computer and Zoom independently. The age threshold of 65 or older was chosen in accordance with widely used conventions in gerontological research and official definitions of older adulthood, such as those adopted by the National Institute on Aging (NIH, 2025). Older adults with mild sensorimotor or perceptual limitations were included in the sample. Exclusion criteria were severe physical or cognitive disabilities and individuals who were unable to use a computer and Zoom independently. Most (77.8%) participants were retired, with an average of 10.71 years in retirement (range 1–23 years). They averaged 15.5 (*SD* = 2.68) years of education. Table 1 presents additional sociodemographic characteristics.

### 2.3. Data Collection

Interviewees were recruited via social media and a word-of-mouth convenience sample and were provided with study information during an initial telephone contact. They signed online informed consent, completed a demographic questionnaire, and received gift certificates as a token of appreciation for their willingness to share their experiences, values, and thoughts. The first author, a certified occupational therapist and PhD student with extensive therapeutic experience, conducted 18 interviews lasting 60 to 90 min. A written semistructured guide developed from relevant literature was used to navigate the interviews and map older adults’ occupational characteristics, needs, preferences, and responses to aging constraints. The interviewer initially posed open-ended questions (Table 2) and transitioned to more focused inquiries as the transcripts were coded. Theoretical sampling occurred by adapting the interview (Butler et al., 2018). We added questions related to the essence of successful aging from the older adult’s viewpoint (“How do you perceive successful aging?”, “What is the difference between a person who ages successfully and one who doesn’t?”, and “What are your dreams for the next years?”). An additional way for theoretical sampling was seeking new characteristics (Butler et al., 2018). After gathering data from 11 interviews, we noticed that the majority described themselves as aging successfully and often expressed strong religious beliefs. To broaden the range of perspectives and enhance theoretical diversity, we sought out additional participants who, during the initial telephone contact explaining the study’s focus on age-related changes and day-to-day functioning, expressed current challenges (e.g., health, social, or functional) and did not identify as religious. After conducting 18 interviews, the categories and subcategories had attained sufficient depth and richness, and additional data no longer altered them, indicating theoretical sufficiency.

### 2.4. Data Analysis

The study was conducted and analyzed with reference to the consolidated criteria for reporting qualitative research (Tong et al., 2007) and standards for reporting qualitative research recommendations (O’Brien et al., 2014). The data were analyzed according to constructivist grounded theory principles, in which the researcher tries to build theories from the data. Constructivist grounded theory is a contemporary revision of the grounded theory, that aims to construct theory through a simultaneous collection and analysis of data. Constructivist grounded theory acknowledges the researchers’ and participants’ multiple standpoints, roles, and realities (Charmaz, 2014, 2017). The researchers used Word and Excel worksheets (version 2019, Microsoft Corporation, Redmond, WA, USA) to perform the coding and analysis process in three phases. In Phase 1, the first author named and coded all data gradually accumulated from the interviews according to their implementation order, resulting in 56 codes (e.g., routine, productivity, optimism, roles). Both transcripts and video recordings were reviewed, allowing the researcher to confirm the content of the transcripts by listening to and viewing the recordings. In Phase 2, the third author, a certified occupational therapist, a PhD student with extensive therapeutic experience, and the last author, a certified PhD occupational therapist, experienced in qualitative and quantitative research, reviewed and confirmed the Phase 1 coding. The three researchers grouped the data around the most commonly used codes. During this phase, the researchers used focused coding, actively selecting the most significant codes to continue toward theoretical conceptualization. For example, in this phase, the researchers encountered multiple recurring codes related to how participants responded to age-related challenges, such as “adjusting to the situation,” “problem-solving,” “accepting limitations,” “adapting to changes,” and “being flexible.” Initially, these codes appeared closely related. However, through the iterative analysis process and constant comparison, it became clear that they reflected two distinct underlying processes. Some codes, such as “accepting limitations” and “adjusting to the situation,” were more passive or attitudinal in nature and thus clustered under the emerging category *acceptance*. Others, such as “problem-solving” and “being flexible,” represented active, intentional efforts to manage difficulties and were grouped under *coping strategies*. This distinction was further sharpened as more interviews were analyzed and the conceptual boundaries between categories became clearer. In addition, several codes referred to behaviors such as “staying active,” “maintaining productivity,” “keeping a daily routine,” and “engaging with others.” At first, the researchers considered these as an additional type of response to age-related changes. However, as the analysis progressed, they came to understand that these behaviors were better conceptualized not as a distinct response, but rather as outcomes of those processes. At this final phase of theoretical coding, the researchers specified relationships among focused codes, consistent with Charmaz’s constructivist approach. Using constant comparison, memo-writing, and diagramming, they linked *personal resources* and *acceptance* as antecedent conditions that enable *coping strategies*, which in turn sustain day-to-day functioning. This process may ultimately lead to successful aging.

Moreover, memos were included in this cyclical process to help construct the three primary categories (Creamer, 2018; Guetterman et al., 2017). The first memos were real-time notes the researcher wrote during the interviews. These memos captured important points and questions (e.g., “feels inferior to others” or “looks on the bright side of life”). Additional memos were produced while reading the transcripts. They involved highlighting important excerpts and assigning initial codes in the margins (e.g., “independence”, “adjustment”). During the process, reflective memos were periodically written to explore emerging relationships between codes. For example, asking, “Does adaptability differ from acceptance?” or “Should ‘meaningful roles’ and ‘self-efficacy’ belong to the same category?”, thereby guiding subsequent analysis. The research team held in-depth discussions about the chosen codes throughout the analysis, and any disagreements were resolved. After reaching a mutual agreement, they shared the initial findings with three participants as part of the validation process. The participants confirmed that the results resonated with their experiences, and no modifications were made based on their feedback. In addition, the emerging categories and subcategories were shared with two colleagues who are experts in gerontology. Their input contributed to refining the phrasing and conceptual clarity of the category labels, particularly those related to *personal resources*.

## 3. Results

**Figure 1:**
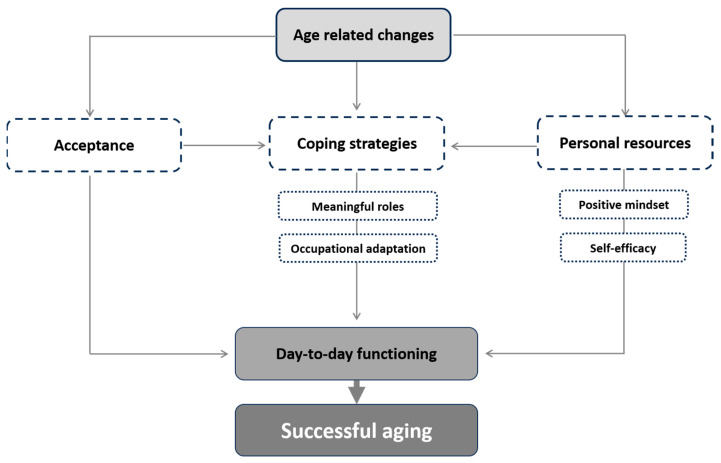
Emerged conceptual model.

Our analysis resulted in a conceptual model (Figure 1) illustrating the dynamic relationships between three core categories regarding older adults’ responses to age-related changes: *acceptance*, *personal resources*, and *coping strategies*. The following sections elaborate on each category in detail.

### 3.1. Acceptance

The *acceptance* concept as a response to aging decline was conveyed through various expressions during the interviews, including accepting and embracing changes that arise with aging. For example, Abigail (age 82 years), a widow who recently lost her husband, shared: “*But I move on. I put a picture of him in the living room and our marriage picture, and I talk to him, …but I go on with life*.” David (74) also expressed acceptance, saying, “*I have friends. Too few, I think, but that’s what I have. Gradually, they are dwindling; there is nothing to do. At this age, some go somewhere else*.”

As individuals age, there is a concurrent decline in mental and physical capacities. Many older adults highlighted their acceptance of these reduced abilities. For example, Natalie (75) described how she copes with new difficulties that arise from aging:

*But you have to accept it, this is how the world is. What can I do? Sometimes, you need to get help. …I wish I could do more, but physically, as time passes, you can do less. …You try to manage with your current abilities.*

Sara (73) shared how she uses singing and music to make herself happy despite the challenges: “*I do everything while singing… Because I know things cannot go back. You cannot be 20 years old again. …There is nothing to do about it*.”

We also identified statements about the ability to accept new situations and challenges independent of the aging context. For instance, Rachel (74) mentioned a challenge and said, “*The two options are to sit on the floor and cry or stand up and move on. I choose the second option.*” Similarly, Sara (73), who faces financial challenges, said,

*People ask me, “How do you smile all the time? You do not have a house of your own; you do not have anything.”’ But I say, “I have health; I have children.” I do not take the walls with me after I die.*

Mia (66), a retired social worker who had to conduct online group sessions, described acceptance in a workplace context: “*Unfortunately, …I learned it. …I hate it; I’m not good at it, and I do not like it, but …you cannot fight it*.” In discussing travel routines, David (74) illustrated another example of coping with inevitable changes:

*I used to travel [abroad] once a year to visit my grandchildren. But now, due to the pandemic, it is over. We do not travel, and we will see …there is nothing to do. They are there. I am here.*

These statements imply that individuals who are used to accepting things they cannot change may adapt to the aging period and its consequences more easily, accept them more flexibly, and stay engaged in various activities despite them.

### 3.2. Personal Resources

The interview analysis revealed that personal resources are a meaningful coping factor. This category comprises two responses to aging decline: *positive mindset* and *self-efficacy*.

#### 3.2.1. Positive Mindset

Although the older adults clearly understood the challenges of aging, many chose to remain optimistic. Hannah (73) explained,

*After all, we are not naïve. We see the age and the years go by, and we see around us …we have lost friends, and we have friends who are very sick. It is quite depressing. …You have to raise your head and look at the positive side. …We have no other choice. Otherwise, we will sink.*

Sara (73) shared, “*We have enough problems, but you need to look at the bright side of life instead of sitting and crying*.” This positive mindset seemed to characterize these older adults since their youth, as Michael (75) noted: “*I am very optimistic by nature. …I try to see the good in everything, not to complain.*” Victoria (74) described another example:

*There are old people who have no one. I always say, thank God, I am in a better situation. …My parents taught us never to cry about our bad fate. Accept the good and accept the bad. And thank God I do not have any bad things for now.*

The ability to look on the bright side of life is particularly evident in stressful situations. Abagail (82) recounted her experiences in a period of social isolation: “*I remember traveling alone, traveling in all kinds of places, seeing flowers in bloom, and then coming home—and that is it. So, it was a kind of ventilation.*” Hannah (73) described the unexpected benefits of a social isolation period: “*A new social circle emerged. …We began gathering with the neighbors on the balcony over the weekends*.”

When asked about their first thoughts in the morning, some participants expressed their general positive attitude toward life. Jacob (66) answered, “*This morning? Good thoughts! I thank God …always, every morning is a good thing. …You should say all the thanks in the world.*” Victoria (74) replied, “*Thank God for waking up and thanks for being able to function.*” They did not take for granted that they were alive and functioning.

Interviewees were asked about the things that disturb or worry them in their daily lives. Ruth (71) replied, “*I am not so disturbed and not so worried. I am quite an optimistic type.*” Mia (66) expanded, “*Actually, I am not really worried. There are only little things [to be worried about]. That is why I told you that the majority is good. There are only small things that bother us.*” Michael (75) summarized these feelings:

*I generally enjoy everything I do. …There are obviously things that you must do. You must pray, eat, do the shopping, and so on. …But it is not a burden for me. …I definitely enjoy going around supermarkets, doing things like that, and shopping. I enjoy everything I do. I more or less enjoy it and see it as neither suffering nor burden.*

These quotations suggest that a positive mindset may lead to better engagement in day-to-day activities like shopping or socializing.

#### 3.2.2. Self-Efficacy

*Self-efficacy* is the second personal resource that may help older adults confront their aging decline. It is defined as a person’s beliefs about their capabilities to organize and perform actions necessary to accomplish specific achievements or desired outcomes (Toledano-González et al., 2018).

It is reasonable to expect that older adults who perceive themselves as more capable and competent will experience fewer difficulties coping as their physical and mental abilities decline. Mia (66), a retired social worker, expressed her feelings about a particular challenge: “*I have the experience of many abilities, and it is okay for me that I do not know everything.*” Jonathan (79) addressed what helps him cope with difficulties in everyday life: *“With what? Which difficulty? I manage everything! I have no difficulties!”*

Ella (68) expressed the importance of feeling competent: “*There is nothing I cannot do. This is how I define myself, and it helps me do things.*” This was also expressed in an opposite example related to Lily’s (69) workplace: “*People really need me and love me when I am there, but I do not feel very competent. So, my first thought [this morning] was that I am not really happy to go there.*” A sense of self-efficacy is important and may be related to better life engagement.

### 3.3. Coping Strategies

The third category of older adults’ responses to the aging challenges, coping strategies, involves the active steps older people take to cope with aging changes more successfully. This category is divided into two subcategories: *meaningful roles* and *occupational adaptation.*

#### 3.3.1. Meaningful Roles

Although not directly asked, many interviewees repeatedly mentioned where they felt needed and could contribute to others. Rachel (74), who goes for medical follow-ups every few months, described:

*I go from one elderly [person] to another and find out [if they need help] …because I also studied a professional medical translation course. I choose the appropriate person and ask if they need help with translation or something else. I encourage the lonely people to talk.*

Many of the older adults found caring for grandchildren meaningful. Mia (66) described:

*My daughter lives next to me with three little children, so they are also part of the routine. …[Some] days I am committed to them. Moreover, I have another daughter in another city with little children, so I travel there once a week.*

The significance of the role of helping others was expressed in many volunteer activities. Victoria (74) said, “*I usually volunteer. I help three elderly families. I have one woman who is a widow and alone.*” David (74) noted, “*The country provided me with opportunities, and I worked and had fun. Now I contribute to others.*” Lily (69) described satisfaction from her role at her former workplace, a mental health care facility: “*I must say, …I received much love from the residents. The satisfaction! Even today, 3 years later, they still call me. So yes, I had tremendous satisfaction.*” Apparently, there was great significance in the idea that a person, despite their advanced age, can contribute to others.

Older adults find various ways to stay active and contribute to their immediate and broader environments. Although not directly asked, many participants shared their meaningful roles within their families and communities, underscoring their significance to them.

#### 3.3.2. Occupational Adaptation

The aging process often leads to changes in routines and activities and raises the need for occupational adaptations. Retirement provides more free time, which some older adults viewed as positive, and others as a burden. Sara (73), who still works part-time, shared:

*I only work in the mornings. …In the afternoon, I participate in various classes. …Some people can work until the middle of the night, but I do not! …This is my choice, and I have no strength for more than that.*

Ruth (71) retired without early preparation:

*I had no crisis. I had no problem with that, and immediately I started participating in classes. …I think it is essential to have management plans; there are so many things to do if you really want to, and you have to plan the time correctly.*

Conversely, some older adults described retirement as emptiness. Lily (69) said,

*I work from 8:00 to 12:00. [Then,] I go to my mother, who is 91 years old, and we have lunch together. …I eat at her place, and sometimes I stay to swim. Sometimes I go home. And then, actually, my main occupation is reading. …I have far too many hours. …I have Pilates classes on the days I do not work. It is nothing, an hour in the day. …It is absolutely nothing.*

These contrasting examples represent the ability or inability to make occupational adaptations as one ages.

After retiring, many older adults find new occupations, like volunteering. David (74) voluntarily taught older adults via Zoom. He said, “*I invest a lot in preparing the lectures and in preparing explanatory videos for each action I teach, so it keeps me busy. I enjoy it and definitely benefit from it. I have no doubts.*” Ben (68) shared, “*Even before I retired, I completed a course to become a social consultant. So did my spouse. Now we voluntarily lead two projects in a nearby community where we facilitate social processes*.” Ben and his spouse proactively planned for this new stage in their lives.

Another example of a new occupation is acquiring an education. Lily (69) shared her satisfaction from learning: “*The truth is that I took a semester at the university, …which is something I really [liked]. It was actually the only thing I really enjoyed!*” David (74) explained, “*You need to move the brain somehow so it does not fall asleep, so I study on average about 2 h a day*.” Lastly, many retired older adults use their free time to care for their grandchildren. Lily (69) said, “*Every second week, the children come on Friday, so I am very busy Thursday and Friday cooking and organizing for it*.”

Similarly, Ruth (71) shared, “*Once a week, we stay with our grandchildren to allow my daughter to work until a later hour. …We make dinner, baths, the whole package*.” Victoria (74) described the benefits of this occupation: “*I cook; I invite the grandchildren. When they come, there is always something to offer them. …It is a pleasure, and that is what keeps me going.*”

Occupational adaptation is also expressed in the *way* older adults perform activities. In this context, David (74) shared an example of the changes that come with age:

*I compare it to the past, when I read a scientific article and knew how to quote every word of it. Today, it is not the same. Today, you have to read it two or three times to remember. The abilities have decreased. There is nothing to do about it.*

Likewise, Natalie (75) explained, “*I do the same things as when I was 30. However, it now takes three times as long.*” Older adults must also adapt to their dependence on others while performing activities. One older adult described her inability to drive at night. Others mentioned needing help with physical activities, such as gardening, housecleaning, climbing a ladder, or opening sinks. Rachel (74) shared her feelings about the lack of complete independence: “*Always, when one of the functions declines, it affects you. It is not so fun to depend on someone else*.” This subcategory emphasizes the implications of aging decline on daily functioning and highlights older adults’ different adaptations and responses.

### 3.4. Emerged Conceptual Model

The analysis suggests a dynamic process in which *acceptance* and *personal resources* may both directly encourage participation in day-to-day activities and create the conditions for using coping strategies. These strategies further support participation in day-to-day activities, a process that may lead to successful aging (see Figure 1).

## 4. Discussion

This interview study expands on concepts initially suggested in our previous study (Tsadok-Cohen et al., 2023). Whereas the previous study addressed successful aging from three viewpoints, those of older adults, their family members, and professionals, the current study’s primary objective was to capture older adults’ insights through in-depth interviews. The interviews revealed older adults’ responses to aging restrictions and deepened our understanding of their relationship with day-to-day functioning and successful aging.

Older adults experience age-related changes and adapt in response to these changes. One example of an adaptive response is *acceptance*, the ability to accept and embrace the changes that come with aging. Our findings suggest that when individuals are accustomed to accepting things they cannot change from a young age, they are likely to adapt to aging and its associated challenges and consequences, and accept it more flexibly. Notably, the capacity for acceptance does not seem to depend on cognitive functions, which often decline with age (Schloss & Haaga, 2011). Thus, acceptance might be an effective regulation strategy for aging populations (Shallcross et al., 2013).

The literature well documents the acceptance concept. Many references discuss acceptance of specific aspects, such as changing technology or self-acceptance of the aging body, and their relation to psychological well-being and successful aging (Jenull et al., 2023; Ozsungur & Hazer, 2020). However, our study underscores the significance of a broader acceptance of day-to-day experiences beyond one’s control. When a person chooses an acceptance response to a challenging situation and “moves on,” it may help them stay actively engaged and age more successfully. The effectiveness of acceptance interventions in physical and mental illness during aging was previously discussed (Kishita et al., 2016). Our study suggests that these interventions, including strategies to help individuals respond more flexibly to challenges and losses, may be adjusted to help older adults without physical or mental illness. For example, an older adult who has lost their spouse might need help accepting the new situation and aging successfully despite the difficulties.

Previous research shows that older adults consider a positive attitude to be a primary construct in successful aging. A positive attitude may help older adults overcome obstacles such as poor health (Reich et al., 2020). Our study also addresses this response to aging declines and stresses that individuals with an optimistic viewpoint on aging have kept a positive attitude toward life situations since their youth. This well-documented understanding underscores optimism as a “stable personality characteristic” with widespread effects across situations (Scheier & Carver, 1985, p. 220). Our findings show that older adults who possess the personal resource of a positive mindset tend to worry less and do not take realities, such as waking up in the morning, for granted. A positive mindset enables better participation in day-to-day activities and greater engagement with life. Interventions should help older adults adopt a more optimistic perspective on aging and focus on the benefits of this stage of life. For instance, older adults could learn how to appreciate the positive aspects of retirement, such as having enough time for leisure activities.

The data analysis also identified *self-efficacy* as an additional personal resource in response to the challenges of aging. This sense of competence tends to peak between the ages of 25 and 65 years, when individuals experience higher levels of activity and engagement in multiple roles. Beyond that age, withdrawal from such roles may lead to reduced feelings of self-efficacy (Mas & Desiderio, 2009).

However, our current study’s data analysis challenges this generalization. Many interviewees reported feeling capable and competent, which helps them cope with the challenges of aging and participate in meaningful activities. This may be explained by the last category in our study, *coping strategies*, and its subcategory of *meaningful roles.* Older adults describe meaningful roles as a core component of health and well-being (Gabriel & Bowling, 2004). Significantly, many participants in our study emphasized their roles and that they can still contribute to others, including caring for grandchildren or volunteering. These findings are consistent with previous studies linking social roles to well-being outcomes through interpretation, including fostering feelings of worth, purpose, or perceptions of usefulness and status (Heaven et al., 2013). Furthermore, maintaining self-defining roles, such as social roles, through active engagement in life is considered a key element of successful aging (Freund & Riediger, 2003).

The second *coping strategies* subcategory is *occupational adaptation*. Klinger (2005) related the process of accepting “the new me” among individuals after traumatic brain injury to occupational adaptation. Her claim also seems relevant to aging populations. To understand the *occupational adaptation* subcategory, one must recognize the crucial role of occupations during aging. A lack of occupation can cause illness, despair, and isolation; the right occupation can promote health and postpone death (Wilcock, 2005).

The term *occupational adaptation* is usually used to address the remediation of underlying cognitive, physical, and affective skills essential for occupational execution and adapting the task or environment to facilitate engagement in occupations (Townsend & Polatajko, 2013). Unlike its usual meaning in populations with disabilities, our study emphasized the significance of *occupational adaptation* in healthy older adults who cope with changes in their abilities and routines. The interviewees expressed the changes they made in occupation types (e.g., volunteering or acquiring education) and intensity (e.g., reduced work hours) to adjust to the new stage of life.

This ability to adjust occupations, rooted in accepting the changed reality and roles (Walder & Molineux, 2017), is probably key to promoting participation in day-to-day activities and aging successfully. Providing occupation-based interventions is a critical issue in health promotion and prevention (Reitz et al., 2020). Occupational adaptation for older adults may be a crucial step toward achieving health promotion values: creating health equity, ensuring social justice, and fostering individual and community empowerment. Occupation-based interventions may include adapting the work environment, developing self-management skills, fall prevention programs, and education and training on activity levels and adaptive coping strategies within daily routines (Reitz et al., 2020; Sendall et al., 2023). Given the crucial role of occupations and activities during aging, the *occupational adaptation* process can contribute to increased participation and more successful aging.

Rowe and Kahn’s (1997) model of successful aging, which seems to be the most widely recognized, primarily refers to older adults free from diseases and disabilities. However, many older adults experience changes in motor, sensory, and cognitive abilities and face multimorbidity (Bay et al., 2023; Harada et al., 2013). Our study’s interviews revealed that older adults often choose to shift their focus away from their diseases and disabilities; instead, they concentrate on their coping strategies and active engagement with life.

These results align with previous research investigating older adults’ subjective perspectives. They found that successful aging’s psychological and social criteria were more important to older adults than biomedical criteria (Reich et al., 2020). In line with our findings, prior research on successful aging showed that older adults do not consistently view disease and disability as signs of unsuccessful aging. Rather, they identify other factors influencing their ability to age successfully, such as resilience, acceptance, engagement with life, and community contribution (Burton et al., 2024).

Being actively engaged and independent is essential to successful aging. Engaging in activities, such as physical, work, volunteer, and leisure activities, is associated with positive health outcomes and life satisfaction among older adults (Burton et al., 2024; Cho & Cheon, 2023; Stav et al., 2012). However, age-related cognitive and physiological changes may reduce involvement in some activities, such as driving and shopping (Falkenstein et al., 2020; Harada et al., 2013). This study supports previous studies that showed older adults can use coping techniques to function and participate better despite the aging restrictions (e.g., Feliciano et al., 2022).

Our study’s results indicate that older adults’ responses to the changes that come with age may enhance their participation in meaningful activities despite the challenges they face. We suggest that the three responses to aging decline, *acceptance*, *personal resources,* and *coping strategies*, might promote day-to-day functioning. Participants with a positive attitude and a sense of competence who accept their aging decline more easily will use coping strategies and therefore be more likely to engage in day-to-day activities, leading to successful aging.

Several limitations to our study should be mentioned. First, the data collection occurred during the COVID-19 pandemic, which may have influenced participants’ attitudes and insights. Nevertheless, the pandemic can be viewed as an example of a stressful situation and various approaches to managing it. Second, since the interviewees were relatively healthy participants recruited through convenience sampling, the emergent conceptual model may over-represent resources and coping patterns and under-represent experiences of frailer older adults who confront different constraints. However, the older adults’ subjective perspectives remain crucial in understanding their coping strategies and responses to aging restrictions and developing suitable interventions. Future research should include participants from diverse cultural backgrounds and with different levels of functional ability. This includes older adults with physical or mental disabilities. Moreover, future studies should use quantitative methods that, combined with this study’s qualitative findings, will provide a more comprehensive understanding of the dynamics between responses to aging restrictions, day-to-day functioning, and successful aging.

## 5. Conclusions

The global aging phenomenon, together with age-related physical and cognitive changes, should spur us to deeply understand the dynamics between older adults’ responses to these changes and day-to-day functioning. Because aging well is subjective and depends on personal views, listening to older adults and uncovering their perspectives is crucial. This study’s findings indicate three types of responses to aging restrictions that appear to contribute to greater engagement in daily life. They may be a key to successful aging among older adults worldwide. Therefore, developing individually tailored interventions for older adults that focus on occupational adaptations according to their needs and preferences is important. As older adults’ health declines, the interventions should focus on maintaining or enhancing coping strategies such as acceptance, a sense of competence, and optimism. These responses to aging restrictions will increase participation in day-to-day functioning and lead to successful aging.

## Figures and Tables

**Table 1:** Participants’ sociodemographic characteristics.

Characteristic	%	*n*
**Gender**		
Female	61.1	11
Male	38.9	7
**Place of birth**		
Israel	44.4	8
Europe	22.2	4
Asia	16.7	3
Africa	5.6	1
Other	11.1	2
**Residence**		
City	61.1	11
Community setting	27.8	5
Village	11.1	2
**Residence type**		
Apartment building	27.8	5
Private house	72.2	13
**Marital status**		
Separated or divorced	16.7	3
Married	66.6	12
Widowed	16.7	3
**Religion affinity**		
Secular	55.6	10
Religious	44.4	8

**Table 2:** Sample key questions from the moderator guide.

Question Number	Question
1	When you think about a typical day, do you have a set routine? Could you please describe a typical day, from getting up in the morning until going to bed?
3	When you think about things in everyday life that you find difficult to do, what helps you do them? How do you tend to react when you fail to perform what you expect of yourself or what the environment expects of you?
5	When you think about your life, what is important to you today? How is your relationship with family members today? With friends? What do you feel you are missing today?
Final	What most disturbs or worries you in your daily life?

## Data Availability

Due to the qualitative nature of the data and ethical restrictions protecting participant confidentiality, the full dataset cannot be made publicly available. However, specific information or excerpts from the data can be shared upon reasonable request, subject to ethical approval.
